# Optimization of Ethanol Extraction Conditions From Sun‐Dried Apricot and Prediction of Antioxidant, Phenolic, and Flavonoid Contents and Their Effects on HCT116 Cells Using Artificial Neural Networks

**DOI:** 10.1002/fsn3.70610

**Published:** 2025-07-17

**Authors:** Kübra Cinar Topcu, Sara Altun Güven, Özlem Cakir, Pınar Anlar, Melekber Sulusoglu Durul, Mizgin Ay, Mohammed Alyafei, Faisal Hayat, Sezai Ercisli

**Affiliations:** ^1^ Aydıntepe Vocational School, Department of Food Processing Bayburt University Bayburt Turkey; ^2^ Faculty of Engineering, Department of Computer Engineering Tarsus University Mersin Turkey; ^3^ Faculty of Engineering, Department of Food Engineering Bayburt University Bayburt Turkey; ^4^ Vocational College of Technical Sciences, Department of Food Processing Atatürk University Erzurum Turkey; ^5^ Agricultural Faculty, Department of Horticulture Kocaeli University Kocaeli Turkey; ^6^ Agricultural Faculty, Department of Horticulture Harran University Sanliurfa Turkey; ^7^ Department of Integrative Agriculture, College of Agriculture and Veterinary Medicine United Arab Emirates University Al Ain UAE; ^8^ Agricultural Faculty, Department of Horticulture Atatürk University Erzurum Turkey; ^9^ HGF Agro, ATA Teknokent Erzurum Turkey

**Keywords:** ANN, antioxidant activity, HCT116, phenolic compounds, RSM, sun‐dried apricot

## Abstract

This study aimed to optimize the ethanol extraction conditions of sun‐dried apricot ( *Prunus armeniaca* L.) and evaluate the antioxidant capacity, phenolic, and flavonoid contents of the obtained extracts. Response surface methodology (RSM) was applied to determine the optimal extraction conditions, which were identified as 60°C temperature, 34% ultrasonic power, 46 min sonication time, and a 4 g/mL solid–liquid ratio. Under these conditions, the extract exhibited a total phenolic content (TPC) of 4.20 mg GAE/g, a total flavonoid content (TFC) of 7.09 mg QE/g, a DPPH (2,2‐diphenyl‐1‐picrylhydrazyl) radical scavenging capacity of 1.37 mg TE/g, a ferric reducing antioxidant power (FRAP) value of 9.12 mg TE/g, and a thiobarbituric acid reactive substances (TBARS) level of 1.69 mg MDA/g. The artificial neural network (ANN) model provided highly accurate predictions for these parameters. Additionally, cell culture experiments demonstrated that the extract exerted a dose‐dependent cytotoxic effect on HCT116 colon cancer cells, significantly reducing their viability. These findings highlight the potential of sun‐dried apricot extracts as natural antioxidants with possible applications in the functional food and pharmaceutical industries.

## Graphical abstract

This study optimized ethanol extraction conditions for dried apricot ( *Prunus armeniaca* L.) using response surface methodology (RSM), achieving optimal yields at 60°C, 34 W, 46 min, and a 4 g/mL solid–liquid ratio. The extract showed high phenolic (4.20 mg GAE/g) and flavonoid (7.09 mg QE/g) content, strong antioxidant capacity, and dose‐dependent cytotoxicity against HCT116 colon cancer cells.

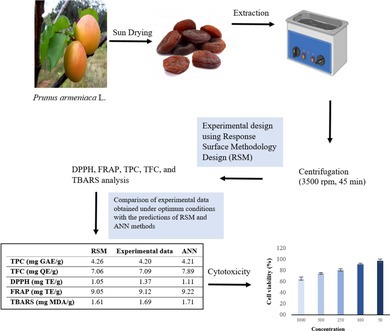

## 1 Introduction

The *Rosaceae* family includes apricots ( *P. armeniaca* ), which naturally grow in Western China, Central Asia, Iran, and the Caucasus region. The global production of commercial apricots occurs mainly in warm temperate zones, and Türkiye stands as a major international leader in this sector (Adachi et al. 2007; Akin et al. 2008; Aamazadeh et al. 2020; Rakida 2023).

Fresh apricot ( *P. armeniaca* ) appears frequently in human diets, and manufacturers produce various products such as jam, juice, marmalade, fruit bars, and dried fruits from this species (Alajil et al. 2021; Khanyile and Gurcan 2024). Although there are many apricot species, *P. armeniaca* is the most commonly cultivated and processed type due to its favorable physicochemical properties (Karatas et al. 2021; Rakida et al. 2023). Sun drying serves as a common preservation technique which extends product shelf life and increases both food fiber composition and nutritional value and antioxidant functionality (Bennett et al. 2011). Sun‐dried apricots have become popular choices for health‐minded consumers seeking nutritious snack options without fat (Devahastin and Niamnuy 2010).

*P. armeniaca* provides essential nutrients, including carbohydrates such as glucose, fructose, and sucrose; vitamins from groups A, C, and E; mineral elements like K, P, Ca, Mg, and Fe; organic acids such as malic and citric acids; and phytochemical components including phenolic compounds and carotenoids (β‐carotene, β‐cryptoxanthin, γ‐carotene) (Moustafa and Cross 2019; Alajil et al. 2021). Traditional medicine has used apricots for treating eye inflammation, as well as infertility and skin conditions and parasitic diseases and spasms (Bousselma et al. 2023; Aktaş Karaçelik 2023). Research has shown that apricot extracts possess antioxidant properties and anti‐inflammatory capabilities as well as antimicrobial effects and possible anticancer potential, which demonstrates their ability to inhibit numerous cancer cell lines (Adachi et al. 2007; Fan et al. 2018; Aamazadeh et al. 2020; Bailly 2020). The bioactive components make apricots a functional food that scientists refer to as the “golden fruit” (Alajil et al. 2021).

The extraction methods used for apricots deeply affect the quantity of bioactive compounds extracted from them. The extraction method, combined with temperature, time, and solvent type, collectively determines the composition and concentration levels of bioactive components from apricots (Ahmed et al. 2023; Aktaş Karaçelik 2023). RSM functions as a standard optimization technology in food processing to measure the combined effects of independent variables on target outcomes (Said and Sarbon 2020; Bhagya Raj and Dash 2022). Another promising modeling approach is artificial neural networks (ANN), which offer superior predictive accuracy, particularly for complex, non‐linear problems (Musa et al. 2016). The food industry shows growing interest in using both technologies, although their practical deployments stay minimal (Said and Sarbon 2020; Alrugaibah et al. 2021; Maselesele et al. 2023).

The main objective of this study was to optimize the extraction conditions of biologically active compounds in sun‐dried apricot ( *P. armeniaca* ) and to evaluate the antioxidant potential and cytotoxic effects of these extracts on HCT116 colon cancer cells. This research contributes to the development of functional food ingredients by identifying apricot extracts with high biologically active compound content and potential health benefits.

## 2 Materials and Methods

### 2.1 Treatment and Extraction of Sun‐Dried Apricot Samples

The apricot fruit ( *P. armeniaca* ) used in this study was harvested from commercial orchards in Malatya, one of the leading apricot‐producing regions of Turkey, between June and July 2023. Fruits were collected from trees grown with traditional agricultural practices. For drying, only apricots of the “Hacıhaliloğlu” variety were preferred due to their suitability in terms of physicochemical properties and widespread use. After harvesting, the fruits were washed with tap water, pitted, and sun‐dried on concrete platforms under open‐air conditions. The dried samples were stored in a cool and dark environment until analysis. Extraction was performed using an ultrasonic‐assisted method (DAIHAN, Ultrasonic cleaner set, WUC‐D06H) with 80% ethanol as the solvent. Modifications following Meng et al. (2011) involved changes in solvent concentration and extraction time. The extracts were centrifuged at 3500 rpm for 45 min and stored at −20°C until analysis.

### 2.2 Experimental Design and Optimization

The extraction conditions were optimized using the Box–Behnken design in Minitab 18.1 software (Table 1). Four independent variables (temperature [*X*_1_, °C], ultrasonic power [*X*_2_, %], sonication time [*X*_3_, min], and solid–liquid ratio [*X*_4_, g/mL]) were evaluated through 27 experimental runs. The effects on DPPH, FRAP, TPC, TFC, and TBARS values were analyzed to maximize antioxidant‐related parameters while minimizing TBARS. Model suitability was assessed via ANOVA, with statistical significance set at *p* < 0.05.

**TABLE 1:** The Box–Behnken design and experimental results were obtained for the measured responses (TPC, TFC, FRAP, DPPH, TBARS).

No.	Temperature (*X*_1_)	Ultrasonic power (%) (*X*_2_)	Sonication time (min) (*X*_3_)	Solid–liquid ratio (*X*_4_)	TPC (mg GAE/g) (*Y*_1_)	TFC (mg QE/g) (*Y*_2_)	DPPH (mg TE/g) (*Y*_3_)	FRAP (mg TE/g) (*Y*_4_)	TBARS (mg MDA/g) (*Y*_5_)
1	30	40	45	4	3.42	3.85	1.02	4.87	1.82
2	30	40	30	8	2.37	4.53	0.87	3.31	2.28
3	30	40	60	8	2.07	2.97	1.07	3.15	2.26
4	30	40	45	12	2.29	3.88	0.55	2.95	2.77
5	30	20	45	8	2.39	4.37	0.83	4.42	2.85
6	30	60	45	8	2.90	5.24	0.89	4.71	2.33
7	45	40	60	4	4.24	7.25	1.03	7.11	2.06
8	45	40	30	4	2.95	6.60	0.82	5.42	1.41
9	45	40	45	8	3.04	6.09	0.90	4.79	2.35
10	45	40	45	8	2.66	6.69	1.00	4.80	2.53
11	45	40	45	8	2.99	5.94	0.99	4.17	2.52
12	45	40	30	12	1.97	5.02	0.60	3.06	2.08
13	45	40	60	12	2.27	4.43	0.85	3.63	3.79
14	45	20	45	4	3.44	7.52	1.12	6.93	1.90
15	45	20	60	8	3.08	6.15	0.93	4.07	3.18
16	45	20	30	8	2.89	5.63	0.84	4.45	2.46
17	45	20	45	12	3.48	6.90	0.70	4.51	3.50
18	45	60	45	4	3.20	5.73	1.04	5.53	1.39
19	45	60	60	8	2.82	5.51	0.74	4.48	3.15
20	45	60	30	8	2.54	4.84	0.74	3.63	2.93
21	45	60	45	12	2.42	4.59	0.32	2.72	3.44
22	60	40	45	4	4.21	9.45	1.09	7.07	1.98
23	60	40	60	8	3.50	7.46	0.61	5.50	2.80
24	60	40	30	8	3.24	6.73	0.98	4.97	2.19
25	60	40	45	12	3.97	7.68	0.33	5.39	3.97
26	60	20	45	8	4.01	6.39	0.92	4.60	2.57
27	60	60	45	8	3.68	7.33	0.81	4.06	2.64

### 2.3 ANN

ANN was employed to model the non‐linear relationships between input parameters (*X*_1_, *X*_2_, *X*_3_, *X*_4_) and output parameters (*Y*_1_: TPC, *Y*_2_: TFC, *Y*_3_: DPPH, *Y*_4_: FRAP, *Y*_5_: TBARS). The dataset used for RSM modeling was also applied to ANN, with 70% allocated for training (20 data points), 15% for validation (5 data points), and 15% for testing (5 data points). The model's predictive capability was enhanced by adjusting the number of layers and neurons in the hidden layer. MATLAB R2021a (64‐bit) and the Neural Network Toolbox were used to develop the feed‐forward ANN model. The best performance was determined based on the highest *R* ^2^ (96.18%) and the lowest RMSE.

### 2.4 Antioxidant and Phenolic Content Analysis

Antioxidant activity was evaluated using DPPH and FRAP assays. For DPPH, 100 μL of extract was mixed with 2000 μL of 0.2 mM DPPH solution, incubated for 30 min, and absorbance was measured at 517 nm. Results were expressed as Trolox equivalents (mg TE/g) (Kumaran and Karunakaran 2007). For FRAP, 150 μL of the extract was reacted with FRAP solution at 37°C for 30 min, and absorbance at 593 nm was recorded, with results expressed as mg TE/g (Upadhyay et al. 2010).

TPC was determined by reacting 1 mL of extract with Folin–Ciocalteu reagent and sodium carbonate. After incubation, absorbance at 750 nm was measured, and TPC was calculated using a Gallic acid standard curve (mg GAE/g) (McDonald et al. 2001).

TFC was measured by mixing 1 mL of extract with 1 mL of 2% AlCl_3_ solution. Absorbance at 430 nm was recorded, and TFC was calculated based on a quercetin standard curve (mg QE/g) (Djeridane et al. 2006).

### 2.5 TBARS

TBARS values were determined by mixing 2 g of the sample with TCA solution, followed by filtration and reaction with TBA solution. After boiling and centrifugation, absorbance was measured at 532 nm. The results were expressed as mg MDA/g (Kilic and Richards 2003).

### 2.6 Cytotoxicity Analysis

The lyophilized extract from sun‐dried apricots was tested for cytotoxicity on HCT116 cells. Cells were cultured in DMEM with FBS, penicillin/streptomycin, and L‐glutamine, and seeded in 96‐well plates. The extract was applied at concentrations of 1000, 500, 250, 100, and 50 μg/mL. After 48 h, cell viability was assessed using CVDK‐8 solution and measured at 450 nm with a microplate spectrophotometer (BioTek, Winooski, VT, USA). Results were calculated relative to control groups.

## 3 Results and Discussion

### 3.1 Optimization of Extraction Conditions

As a result of the analyses, it was found that the methods used for the determination of phenolic compounds, flavonoids, and antioxidant activities were in high agreement and yielded satisfactory *R* ^2^ values. The *R* ^2^ values for TPC, TFC, FRAP, DPPH, and TBARS analyses were 86.03%, 81.36%, 81.01%, 82.10%, and 85.95%, respectively (Table 2).

**TABLE 2:** Quadratic equations were obtained as a result of regression analysis.

Responses	Model	*R* ^2^	Lack of Fit
TPC (mg GAE/g)	*Y*_1_ = 2.15 − 0.0906 *X*_1_** + 0.0205 *X*_2_ + 0.1177 *X*_3_ − 0.213 *X*_4_ + 0.001119 *X*_1_** × *X*_1_** + 0.000259 *X*_2_ × *X*_2_ − 0.001161*X*_3_ × *X*_3_ + 0.01435 *X*_4_** × *X*_4_** − 0.000697 *X*_1_** × *X*_2_ + 0.000620 *X*_1_** × *X*_3_ + 0.00370 *X*_1_** × *X*_4_** + 0.000080 *X*_2_ × *X*_3_ − 0.00257 *X*_2_ × *X*_4_** − 0.00414 *X*_3_ × *X*_4_**	86.03%	0.259
TFC (mg QE/g)	*Y*_2_ = 3.85 + 0.040 *X*_1_** + 0.006 *X*_2_ + 0.098 *X*_3_−0.592 *X*_4_** − 0.00010 *X*_1_** × *X*_1_** − 0.000338*X*_2_ × *X*_2_ − 0.00134 *X*_3_ × *X*_3_ + 0.0313 *X*_4_** × *X*_4_** − 0.00069 *X*_1_** × *X*_2_ + 0.00078 *X*_1_** × *X*_3_ + 0.00097 *X*_1_** × *X*_4_** + 0.00102 *X*_2_ × *X*_3_ − 0.00124 *X*_2_ × *X*_4_** − 0.00463 *X*_3_ × *X*_4_**	81.36%	0.180
DPPH (mg TE/g)	*Y*_3_ = 2.40 + 0.0642 *X*_1_ + 0.0274 *X*_2_ + 0.0489 *X*_3_ + 0.1458 *X*_4_** − 0.000261 *X*_1_ × *X*_1_ − 0.000174 *X*_2_ × *X*_2_ 0.000189 *X*_3_ × *X*_3_ − 0.00745 *X*_4_** × *X*_4_** − 0.000142 *X*_1_ × *X*_2_ − 0.000629 *X*_1_ × *X*_3_ − 0.00119 *X*_1_ × *X*_4_ − 0.000066 *X*_2_ × *X*_3_ − 0.000929 *X*_2_ × *X*_4_** + 0.00016 *X*_3_ × *X*_4_**	81.01%	0.125
FRAP (mg TE/g)	*Y*_4_ = −3.72 + 0.143 *X*_1_** + 0.025 *X*_2_ + 0.150 *X*_3_ + 0.310 *X*_4_** − 0.00098 *X*_1_** × *X*_1_** − 0.000448 *X*_2_ × *X*_2_ − 0.00251 *X*_3_ × *X*_3_ + 0.0099 *X*_4_** × *X*_4_** + 0.00006 *X*_1_** × *X*_2_ + 0.00255 *X*_1_** × *X*_3_ − 0.00747 *X*_1_** × *X*_4_** + 0.00012*X*_2_ × *X*_3_ − 0.00161 *X*_2_ × *X*_4_** − 0.00517 *X*_3_ × *X*_4_**	82.10%	0.149
TBARS (mg MDA/g)	*Y*_5_ = 5.75−0.0770 *X*_1_−0.0644 *X*_2_−0.0356 *X*_3_** − 0.220 *X*_4_** + 0.000007 *X*_1_ × *X*_1_ + 0.000590 *X*_2_ × *X*_2_ + 0.000076*X*_3_** × *X*_3_** − 0.00275 *X*_4_** × *X*_4_** + 0.000500 *X*_1_ × *X*_2_ + 0.000707*X*_1_ × *X*_3_** + 0.00435 *X*_1_ × *X*_4_** − 0.000422 *X*_2_ × *X*_3_** + 0.00140 *X*_2_ × *X*_4_** + 0.00442 *X*_3_** × *X*_4_**	85.95%	0.064

*Note:* The term is significant at **p ≤ 0.05, (*X*_1_, temperature [°C]; *X*_2_, ultrasonic power [%]; *X*_3_, sonication time [min]; *X*_4_, solid–liquid ratio [g/mL]).

### 3.2 Effect of Experimental Conditions on TPC, TFC, FRAP, DPPH, and TBARS

**FIGURE 1:**
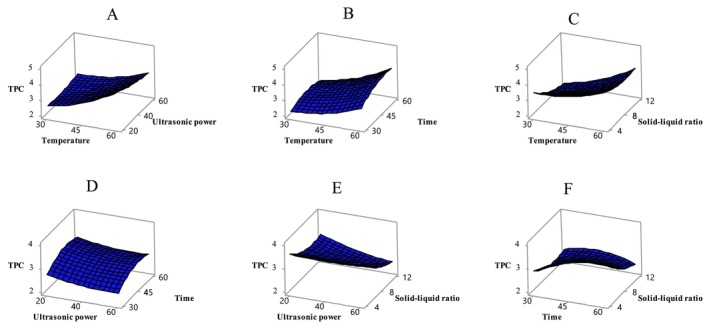
Variation of TPC value with independent variables temperature‐ultrasonic power (A), temperature–time (B), temperature‐solid–liquid ratio (C), ultrasonic power‐time (D), ultrasonic power‐solid–liquid ratio (E), time‐solid–liquid ratio (F). Temperature (*X*_1_, °C), ultrasonic power (*X*_2_, %), time (*X*_3_, min), and solid–liquid ratio (*X*_4_, g/mL).

The statistical evaluation of the experimental design on the dependent variables (Table 3) indicates that the model is significant in terms of TPC (*p* < 0.05). Among the independent variables, temperature and solid–liquid ratio were found to have a particularly significant effect on TPC (*p* < 0.05). The effect of these independent variables on TPC is illustrated in Figure 1. In the studies on apricot, it has been determined that the total phenolic amount varies depending on various factors (apricot variety, applied process, extraction conditions, etc.). The phenolic content of sun‐dried apricots, which were also used in the present study, was rather high and was determined in the range of 1.97–4.24 mg GAE/g. In one study, the total phenolic content ranged from 25.31 to 89.95 mg GAE/100 g across 6 different (CITH‐A‐1, CITH‐A‐2, CITH‐A‐3, Roxana, Gold Cot, and Shakarpara) apricot genotypes (Alajil et al. 2021). Previous studies showed that 11 Turkish apricot (Hacıhaliloğlu, Hasanbey, Soğancı, Kabaaşı, Çataloğlu, Cöloğlu, Hacıkız, Tokaloğlu, Alyanak, Iğdır, and Bursa) varieties contained total phenolic matter ranging from 4233 to 8180 mg GAE/100 g dry matter (Akin et al. 2008). A study examined the bioactive properties of extracts extracted from both fresh and dried apricots gathered from four different apricot types (Zerdali, Teberze, Şalak, and Ordubat). Various evaluations determined Zerdali possessed the highest level of total phenolic components across all tested varieties (456.482 ± 4.243 mg GAE/100 g). In addition, the study found that dried apricots extracted with methanol had higher phenolic content than fresh apricots extracted with water (Aktaş Karaçelik 2023). Solvent extraction effectiveness with phenolic compounds seems to depend on dielectric constant measurements. The polarity of the solvent adjusts based on the constant's numerical value. Under normal conditions, as this constant increases, the polarity of the sample increases, and its dissolving power also rises. Accordingly, pure methanol is considered to provide higher extraction efficiency than ethanol and water (Bosso et al. 2016).

**TABLE 3:** ANOVA statistics for the experimental design of dependent variables (TPC, TFC, FRAP, DPPH, TBARS).

	TPC		TFC		DPPH		FRAP		TBARS	
	*F*	*p*	*F*	*p*	*F*	*p*	*F*	*p*	*F*	*p*
Model	5.28	0.003	3.74	0.014	3.66	0.015	3.93	0.011	5.24	0.003
Linear	13.91	0.000	11.35	0.000	9.85	0.001	12.22	0.000	15.92	0.000
*X*_1_	34.01	0.000	9.91	0.008	1.14	0.306	41.16	0.000	2.17	0.167
*X*_2_	1.95	0.188	2.20	0.164	2.93	0.112	1.40	0.260	0.22	0.645
*X*_3_	2.68	0.127	1.43	0.255	0.68	0.425	0.02	0.896	9.66	0.009
*X*_4_	17.01	0.001	31.88	0.000	34.66	0.000	6.30	0.027	51.64	0.000
Square	2.83	0.073	1.28	0.331	1.13	0.387	0.77	0.567	0.78	0.562
*X*_1_*X_1_	2.69	0.127	0.01	0.943	1.01	0.335	0.32	0.585	0.00	0.992
*X*_2_*X_2_	0.46	0.512	0.17	0.684	1.42	0.256	0.21	0.657	2.28	0.157
*X*_3_*X_3_	2.90	0.114	0.86	0.373	0.53	0.479	2.06	0.177	0.01	0.915
*X*_4_*X_4_	2.24	0.160	2.38	0.149	4.17	0.064	0.16	0.694	0.08	0.783
Interaction	1.15	0.391	0.31	0.921	1.21	0.365	0.52	0.783	1.10	0.416
*X*_1_*X_2_	1.40	0.260	0.31	0.589	0.40	0.540	0.00	0.969	0.69	0.422
*X*_1_*X_3_	0.62	0.446	0.22	0.648	4.41	0.057	1.59	0.231	0.78	0.395
*X*_1_*X_4_	1.57	0.233	0.02	0.880	1.13	0.309	0.97	0.343	2.09	0.174
*X*_2_*X_3_	0.02	0.895	0.67	0.428	0.09	0.774	0.01	0.938	0.49	0.497
*X*_2_*X_4_	1.35	0.268	0.07	0.796	1.21	0.292	0.08	0.781	0.39	0.545
*X*_3_*X_4_	1.97	0.186	0.55	0.473	0.02	0.891	0.47	0.508	2.16	0.167

*Note:* The term as, *X*_1_, temperature (°C); *X*_2_, ultrasonic power (%); *X*_3_, sonication time (min); *X*_4_, solid–liquid ratio (g/mL).

**FIGURE 2:**
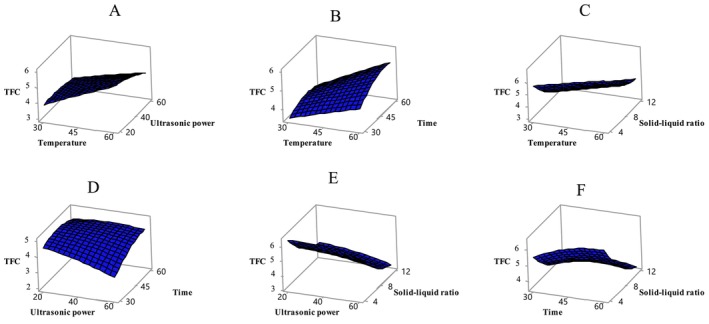
Variation of TFC value with independent variables temperature‐ultrasonic power (A), temperature–time (B), temperature‐solid–liquid ratio (C), ultrasonic power‐time (D), ultrasonic power‐solid–liquid ratio (E), time‐solid–liquid ratio (F). Temperature (*X*_1_, °C), ultrasonic power (*X*_2_, %), time (*X*_3_, min), and solid–liquid ratio (*X*_4_, g/mL).

The model also has a significant effect on TFC (*p* < 0.05) (Table 3). TFC varied significantly under the tested conditions, with temperature and solid–liquid ratio showing the most pronounced effects (*p* < 0.05). Figure 2 illustrates the effects of these variables on TFC values. In this study, the TFC values of the extracts ranged from 2.97 to 9.45 mg QE/g. Literature comparisons indicate that flavonoid content in apricots varies considerably. Flavonols, a subgroup of flavonoids, were identified in apricot, one of 28 different fruits collected from local markets. In the study, the flavonol content of apricot was reported as 4.65 mg per 100 g of dry weight (García‐Alonso et al. 2004). Another study found that the total flavonoid content in different apricot varieties (CITH‐A‐1, CITH‐A‐2, CITH‐A‐3, Roxana, Gold Cot, and Shakarpara) ranged from 7.71 to 15.46 mg CE/100 g (Alajil et al. 2021).

**FIGURE 3:**
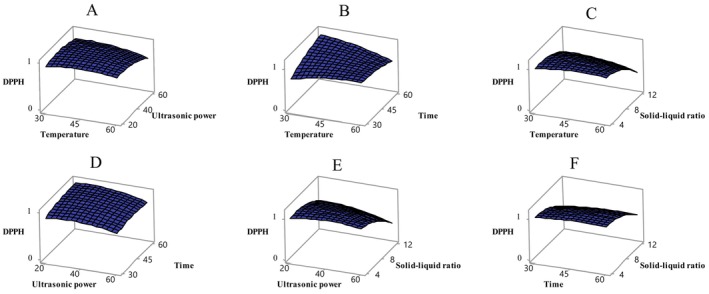
Variation of DPPH value with independent variables temperature‐ultrasonic power (A), temperature–time (B), temperature‐solid–liquid ratio (C), ultrasonic power‐time (D), ultrasonic power‐solid–liquid ratio (E), time‐solid–liquid ratio (F). Temperature (*X*_1_, ^o^C), ultrasonic power (*X*_2_, %), time (*X*_3_, min), and solid–liquid ratio (*X*_4_, g/mL).

**FIGURE 4:**
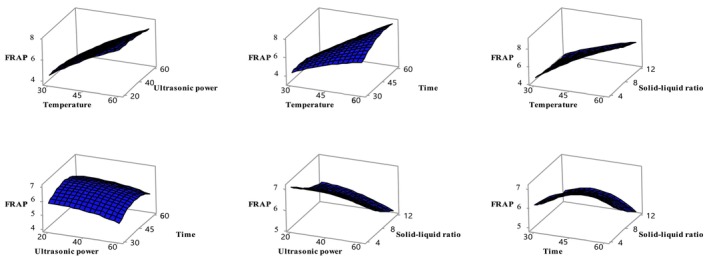
Variation of FRAP value with independent variables temperature‐ultrasonic power (A), temperature–time (B), temperature‐solid–liquid ratio (C), ultrasonic power‐time (D), ultrasonic power‐solid–liquid ratio (E), time‐solid–liquid ratio (F). Temperature (*X*_1_, °C), ultrasonic power (*X*_2_, %), time (*X*_3_, min), and solid–liquid ratio (*X*_4_, g/mL).

Antioxidant activity was evaluated using the DPPH and FRAP methods in ethanol extracts obtained from sun‐dried apricot samples. Table 3 shows that the independent variables had a significant effect on both indicators of antioxidant activity (*p* < 0.05). In particular, the solid–liquid ratio significantly affected DPPH activity, while temperature and solid–liquid ratio significantly influenced FRAP activity (*p* < 0.05). The relationships between these variables and antioxidant activity are shown in Figures 3 and 4. The Box–Behnken design analysis revealed that DPPH and FRAP activities in sun‐dried apricot samples varied depending on the applied conditions. The effects of different temperatures (4, 15°C, 25°C, and 35°C), packaging types (vacuum and normal), and light/dark conditions on the quality changes of dried apricots during 6 months of storage were examined. The study found that as the temperature increased from 4°C to 25°C, DPPH and FRAP values decreased, but both increased again at 35°C. Vacuum packaging and dark storage had no significant effect on antioxidant capacity (Deng et al. 2022). In another study, the peel and pulp of six different apricot varieties (Xiaobai, Liguang, Katy, Chuanzhihong, Dajie, and Shushanggan) were ultrasonically extracted using 70% ethanol. It was determined that the peels of all apricot varieties exhibited higher antioxidant activity than the pulp (Fan et al. 2018). However, the FRAP content of freeze‐dried apricot fruit was found to be 39.6 ± 6.4 μmol Fe^2+^/g dried fruit (Bennett et al. 2011).

**FIGURE 5:**
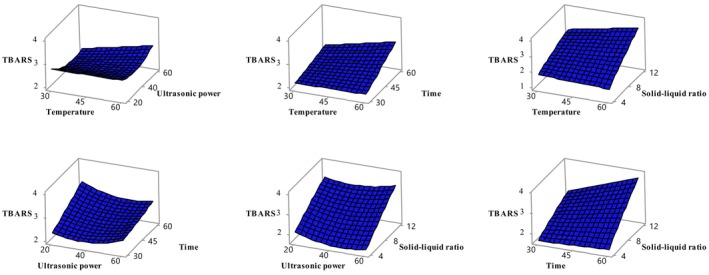
Variation of TBARS value with independent variables temperature‐ultrasonic power (A), temperature–time (B), temperature‐solid–liquid ratio (C), ultrasonic power‐time (D), ultrasonic power‐solid–liquid ratio (E), time‐solid–liquid ratio (F). Temperature (*X*_1_, °C), ultrasonic power (*X*_2_, %), time (*X*_3_, min), and solid–liquid ratio (*X*_4_, g/mL).

Lipid oxidation was assessed using TBARS values, and sonication time and solid–liquid ratio were found to have statistically significant effects (*p* < 0.05) (Table 3). Figure 5 illustrates these effects, highlighting the importance of optimizing these parameters to reduce lipid oxidation. In the previous study, the TBARS value in apricot fruit was determined as 900 μg among 28 different fruits (García‐Alonso et al. 2004). Given the limited number of studies on TBARS determination in apricot fruit, the present study is anticipated to be a pioneering contribution to the field.

In general, this study revealed that extraction temperature, solid–liquid ratio, and sonication time had significant effects on TPC, TFC, antioxidant activity, and lipid oxidation levels in sun‐dried apricots ( *P. armeniaca* ). In future studies, it is recommended to investigate the mechanisms underlying these effects in more detail and to evaluate the effects of different apricot varieties and post‐harvest processing techniques.

### 3.3 Comparison of Optimum Conditions Using RSM and ANN With Experimental Data

Although ANN and RSM are two different modeling techniques, the application of ANN in food processing is relatively newer than RSM (Bhagya Raj and Dash 2022). Nevertheless, ANN has been employed in various applications, including food image analysis, thermal and non‐thermal food processing operations modeling, food safety and quality analysis (Goyal and Goyal 2011; Ramzi et al. 2015; Simić et al. 2016). However, no studies were found in the literature that optimized the extraction of dried apricot fruits and compared the results using RSM and ANN methods. In the present study, the optimal conditions were determined to be a temperature of 60°C, an ultrasonic power of 34%, a sonication time of 46 min, and a solid–liquid ratio of 4 g/mL. Under these conditions, the estimated TPC, TFC, DPPH, FRAP, and TBARS values obtained from the model were 4.26 mg GAE/g, 7.06 mg QE/g, 1.05 mg TE/g, 9.05 mg TE/g, and 1.61 mg MDA/g, respectively. The experimentally obtained results were 4.20 mg GAE/g, 7.09 mg QE/g, 1.37 mg TE/g and 9.12 mg TE/g, 1.69 mg MDA/g, respectively. In comparison, the values estimated by ANN were 4.21 mg GAE/g for TPC, 7.89 mg QE/g for TFC, 1.11 mg TE/g for DPPH, 9.22 mg TE/g for FRAP, and 1.71 mg MDA/g for TBARS. From these results, it was observed that the ANN and RSM predictions and experimental data were very compatible with each other (Table 4).

**TABLE 4:** Comparison of experimental data obtained under optimum conditions with the predictions of RSM and ANN methods.

	RSM	Experimental data	ANN
TPC (mg GAE/g)	4.26	4.20	4.21
TFC (mg QE/g)	7.06	7.09	7.89
DPPH (mg TE/g)	1.05	1.37	1.11
FRAP (mg TE/g)	9.05	9.12	9.22
TBARS (mg MDA/g)	1.61	1.69	1.71

In conclusion, both ANN and RSM methods stand out as effective modeling tools. However, ANN may provide more accurate estimates, especially in cases where more complex and nonlinear relationships are present. However, considering the lack of statistical validation and gaps in the literature, it is recommended to use statistical tests such as RMSE and *R* ^2^ to more reliably evaluate the performance of both models in future studies. In addition, the advantages and disadvantages of ANN should be further investigated, especially in food extractions, and validated with more samples.

### 3.4 Cytotoxicity

**FIGURE 6:**
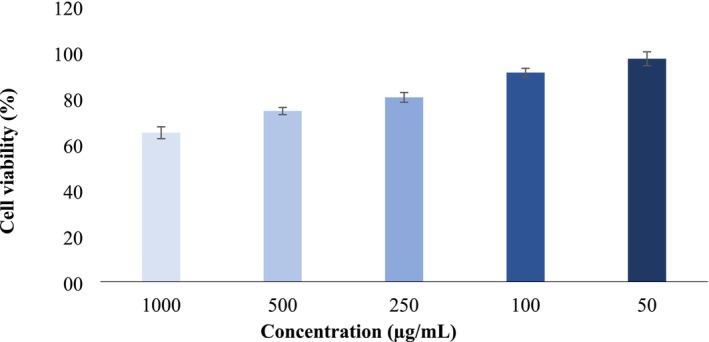
Cytotoxic effect of ethanol extract of sun‐dried apricot under optimum conditions on HCT116 cell line after 48 h. Mean ± SD.

Cancer, one of the leading causes of death today, is typically treated with surgery and chemotherapy. However, these methods may be ineffective in advanced stages of cancer and can cause significant side effects. Therefore, in recent years, interest in substances with natural anti‐cancer properties has increased (Adachi et al. 2007; Aamazadeh et al. 2020; Hu et al. 2023). Fruits are often regarded as nature's true medicines, and research in this area is also of significant importance (Nishi et al. 2020). In this context, determining the cytotoxic effect of optimized sun‐dried apricot extract on the HCT116 colon cancer cell line is important, as colon cancer is one of the most prevalent cancer types worldwide. Colon cancer affects both men and women and is significantly influenced by nutrition (Cassiem and de Kock 2019). For this reason, in the current study, the cytotoxic effect of ethanol extract from dried apricot fruit under optimized conditions on HCT116 cells was investigated after 48 h. The results indicated a decrease in cell viability with increasing concentration (Figure 6). Although no studies in the literature have investigated the effect of dried apricot extract under optimized conditions on colon cancer, there are studies on colon cancer involving extracts from various apricot varieties obtained through different methods (Mori et al. 2007; Cassiem and de Kock 2019; Nishi et al. 2020; Keleştemur et al. 2024). Similar to the current research, previous research has reported that apricot extracts reduced the survival rate of colon cancer cells.

## 4 Conclusions

This research evaluated the antioxidant properties and total phenolic compounds and flavonoid content, and TBARS value of sun‐dried apricots using an optimized extraction method based on RSM. Under the optimized conditions, the fruit extract displayed maximum content of antioxidants as well as total phenolics and flavonoids, together with minimal TBARS value. The extract containing the highest bioactive components showed cytotoxic effects on HCT116 cells, where cell viability decreased with increasing concentrations. The results revealed that the ANN prediction method for DPPH, FRAP, TPC, TFC, and TBARS exhibited excellent accuracy in matching experimental results and RSM model predictions. The optimized dried apricot extracts exhibited both strong antioxidant properties and significant cytotoxic killing ability against HCT116 cells. The prediction accuracy of the ANN model proves that this model is appropriate for process optimization purposes. The research findings demonstrate how apricot extracts can become applicable for functional food development alongside pharmaceutical applications. The combination of ANN and RSM methods for dried apricot extract preparation and cytotoxic effect determination under optimal conditions remains unpublished in previous scientific works. This suggests a substantial contribution.

## Author Contributions

**Kübra Cinar Topcu:** methodology (equal), formal analysis (equal), visualization (equal), writing – review and editing (equal). **Sara Altun Güven:** methodology (equal), software (equal). **Özlem Cakir:** formal analysis (equal), writing – review and editing (equal). **Pınar Anlar:** methodology (equal), writing – original draft (equal), writing – review and editing (equal). **Melekber Sulusoglu Durul:** writing – review and editing (equal). **Mizgin Ay:** writing – review and editing (equal). **Mohammed Alyafei:** writing – review and editing (equal). **Faisal Hayat:** writing – review and editing (equal). **Sezai Ercisli:** writing – review and editing (equal).

## Conflicts of Interest

The authors declare no conflicts of interest.

## Data Availability

Data supporting the findings of this study are available on request from the corresponding author.
